# Perception of the relationship between TMD and orthodontic treatment among orthodontists

**DOI:** 10.1590/2176-9451.20.1.045-051.oar

**Published:** 2015

**Authors:** Thaís Gonzalez da Silveira Coêlho, Hugo Cesar Pinto Marques Caracas

**Affiliations:** 1 Professor, Catholic University of Brasília (UCB); 2 PhD in Orthodontics, Federal University of Rio de Janeiro (UFRJ)

**Keywords:** Temporomandibular joint disorders, Orthodontic appliances, Orthodontics

## Abstract

**INTRODUCTION:**

The consensus about the relationship between TMD and orthodontic treatment has gone from a cause and effect association between TMD and orthodontic treatment to the idea that there is no reliable evidence supporting this statement.

**OBJECTIVE:**

To assess the beliefs, despite scientific evidence, of Brazilian orthodontists about the relationship between TMD and orthodontic treatment with regards to treatment, prevention and etiology of TMD.

**METHODS:**

A survey about the relationship between TMD and orthodontic treatment was prepared and sent to Brazilian orthodontists by e-mail and social networks. Answers were treated by means of descriptive statistics and strong associations between variables were assessed by qui-square test.

**RESULTS:**

The majority of orthodontists believe that orthodontic treatment not only is not the best treatment option for TMD, but also is not able to prevent TMD. Nevertheless, the majority of orthodontists believe that orthodontic treatment can cause TMD symptoms.

**CONCLUSION:**

This study suggests that orthodontists' beliefs about the relationship between orthodontic treatment and TMD are in accordance with scientific evidence only when referring to treatment and prevention of TMD. The majority of orthodontists believe that, despite scientific evidence, orthodontic treatment can cause TMD.

## Abstract (pt)

**INTRODUÇÃO:**

o consenso sobre a relação entre DTM e tratamento ortodôntico foi de uma associação de causa e efeito à ideia de que não há evidências confiáveis que suportem essa afirmação.

**OBJETIVO:**

avaliar as crenças, sem considerar as evidências, de ortodontistas brasileiros sobre a relação entre DTM e tratamento ortodôntico com relação ao tratamento, prevenção e etiologia da DTM.

**MÉTODOS:**

um questionário sobre a relação entre DTM e tratamento ortodôntico foi preparado e enviado a ortodontistas brasileiros por meio de e-mail e mídias sociais. As respostas foram analisadas por estatística descritiva, e fortes associações entre as variáveis foram verificadas pelo teste χ^2^.

**RESULTADOS:**

a maioria dos ortodontistas acredita que o tratamento ortodôntico não é o melhor tratamento para DTM. Além disso, acreditam que não é a melhor forma para sua prevenção. Também, a maioria dos ortodontistas acredita que o tratamento ortodôntico pode causar sintomas de DTM.

**CONCLUSÃO:**

este estudo sugere que as crenças dos ortodontistas sobre a relação entre tratamento ortodôntico e DTM estão de acordo com as evidências científicas apenas quando se trata do tratamento e da prevenção de DTM. A maioria dos ortodontistas acredita que, apesar das evidências científicas, o tratamento ortodôntico pode causar DTM.

## INTRODUCTION

According to the American Academy of Orofacial Pain, temporomandibular disorder (TMD) is a collective term that embraces a number of clinical problems involving the masticatory muscles and/or associated structures such as the temporomandibular joint (TMJ), and may go beyond pathologies involving TMJ.^1^ The term does not refer to a single entity, since many diseases with large variations regarding the anatomic location, clinical characteristics, etiology and progression, are within this single denomination.^2^

Signs and symptoms such as pain, limited opening, asymmetrical movement of the jaw, and joint sounds are the most common findings.^3^ Some signs and symptoms of TMD also occur in healthy individuals, with prevalence concentrated in the group between 15 and 25 years-old.^4^ They have a tendency to increase with age,^5^ until the fifth decade of life^6^ when a reduction in prevalence is observed. It is estimated that 37.5% of the overall population has had at least one symptom of TMD, whereas approximately 41.3% to 68.6% of university students have signs or symptoms of TMD.^7^ Prevalence is higher among women possibly due to estrogen action; however, additional studies are warranted to further confirm this statement.^8^^,^ 9

Over the years, the relationship established between dental occlusion and TMD, or orthodontic treatment and TMD has been the subject of numerous investigations, drawing opposite conclusions. Several studies have pointed out that occlusion is important for the development of TMD,^10^ and that orthodontic treatment has some influence over the signs and symptoms of TMD.^11^ Moreover, extensive reviews and clinical studies do not indicate that orthodontic treatment predisposes^12^^-^ 15 or decreases the risk of developing future TMD.^16^^-^ 20 Furthermore, orthodontic treatment has not been indicated as initial therapy for patients with TMD.^13^^,^ 20 ^-^ 23 Recent studies highlight that occlusion-changing procedures are no longer considered appropriate for most patients with TMD.^24^ In fact, significant scientific evidence points out a trend of non-association between orthodontic treatment, occlusion and temporomandibular disorder.^25^

The aim of this study is to assess the beliefs, despite scientific evidence, of Brazilian orthodontists about the relationship between TMD and Orthodontics with regard to treatment, prevention and etiology of TMD.

## MATERIAL AND METHODS

A questionnaire was developed and initially sent to Brazilian orthodontists members of the Brazilian Association of Orthodontics (ABOR). The questionnaire was sent by e-mail along with an explanatory message and a link that redirected the participant to the website. The link was also published on social networks along with an explanatory message requesting respondents to forward it to his/her contact list so that the initial number of participants could be increased. Participants were free to get in touch with the authors in case of doubts while answering the questionnaire. The first e-mails were sent in October 2012, and each respondent was encouraged to forward the initial e-mail to his/her contact list. The deadline was January 13^th^, 2013. Thus, the survey took three months.

**Figure 1 -:**
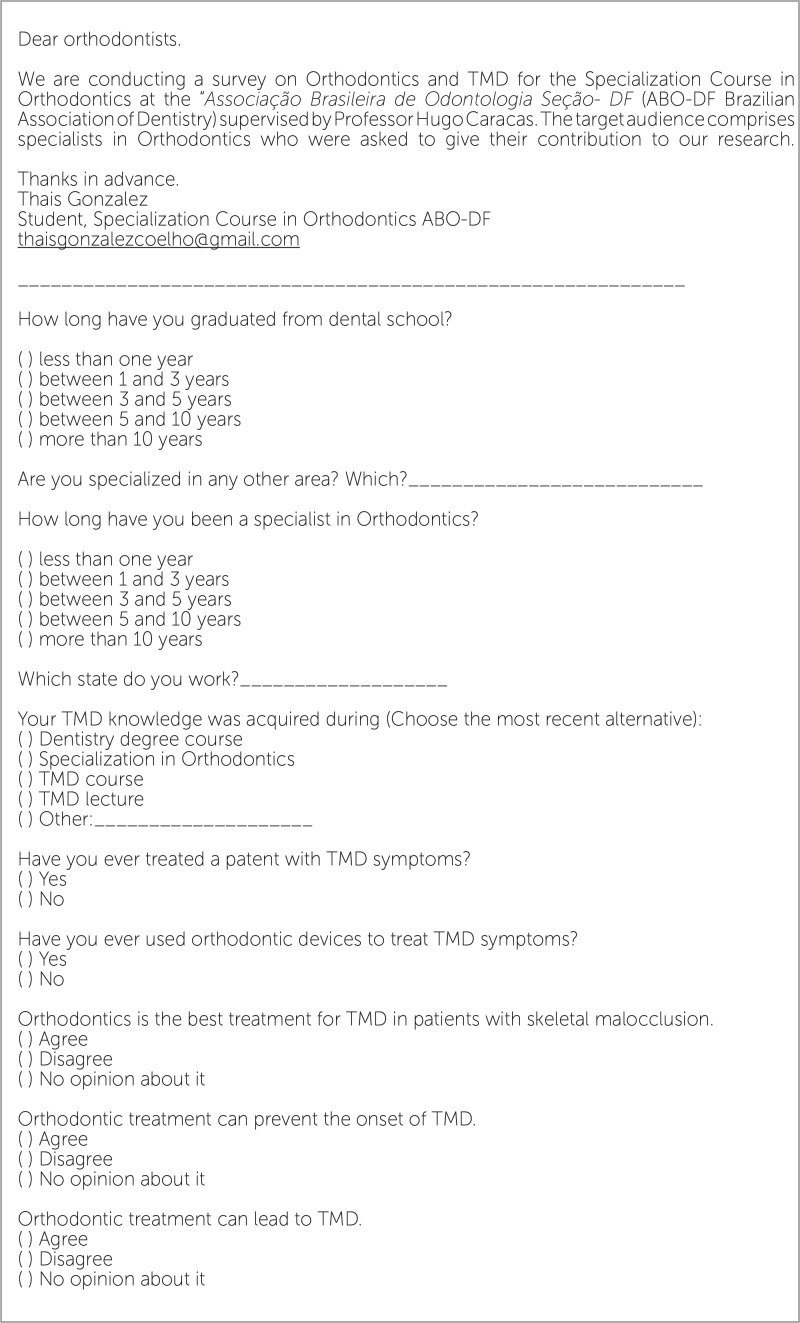
Questionnaire sent to orthodontists.

The questionnaire comprised questions aimed to collect basic information about each participant and questions about the respondent's beliefs and clinical management of patients with TMD symptoms (Fig 1), including: The time since graduation in Dentistry and Orthodontics; the state where the orthodontist works; whether he/she has a postgraduate degree in some other area; and where TMD knowledge had been acquired. Two other questions were asked with regards to the clinical routine of the professional: "Have you ever treated a patient with TMD symptoms?" and "Have you ever used orthodontic devices to treat TMD symptoms?".

Subsequently, three statements, to which the participant could agree, disagree, or have no opinion, were made: "Orthodontics is the best treatment for TMD in patients with skeletal malocclusion"; "Orthodontic treatment can prevent the onset of TMD"; and "Orthodontic treatment can lead to TMD".

After the deadline, descriptive statistics was used to analyze the answers, and chi-square test was performed to assess associations between variables.

## RESULTS

After three months, 173 answers were received. Distribution according to how long orthodontists had graduated from dental school is summarized inTable 1 together with distribution of opinions about the three statements. No relationship was established between the time since graduation and the answers given. Likewise, no statistically significant association was found between statements and time of graduation in Dentistry. Answers of "less than one year after graduation" in Dentistry were excluded, since this study included orthodontists, only. In other words, dentists who had concluded the Dentistry course in less than one year would not have completed their studies in Orthodontics, as it lasts at least two years.

**Table 1 -:** Distribution of data according to how long orthodontics had graduated from dental school and participants' opinion about the three statements.

	Answer	How long have you graduated from dental school?				Total
		From 1 to 3 years	From 3 to 5 years	From 5 to 10 years	More than 10 years	
Orthodontics is the best treatment for TMD patients with skeletal malocclusion	Disagree	4	11	25	102	142
	Agree	0	1	4	16	21
	No opinion	0	0	3	7	10
	Total	4	12	32	125	173
Orthodontic treatment can prevent the onset of TMD	Disagree	2	6	20	75	103
	Agree	2	6	11	46	65
	No opinion	0	0	1	4	5
	Total	4	12	32	125	173
Orthodontic treatment can lead to TMD	Disagree	1	6	8	61	76
	Agree	2	4	23	61	90
	No opinion	1	2	1	3	7
	Total	4	12	32	125	173

Thus, the final sample comprised orthodontists, only; of which 6.9% had been specialists for less than one year, 11% for 1 to 3 years, 10.4% for 3 to 5 years, 23.1% for 5 to 10 years, and 48.6% for more than 10 years.

Answers given on additional postgraduate degrees were divided into three groups: specialists in Orthodontics, only, accounted for 67.6%; specialists in Orthodontics and TMD accounted for 8.7%; and specialists in Orthodontics and another specialty accounted for 23.7%.

Most answers were given by orthodontists who lived in the Federal District, followed by São Paulo and Goiânia. Some of them were also obtained from overseas, as illustrated inFigure 2.

**Figure 2 -:**
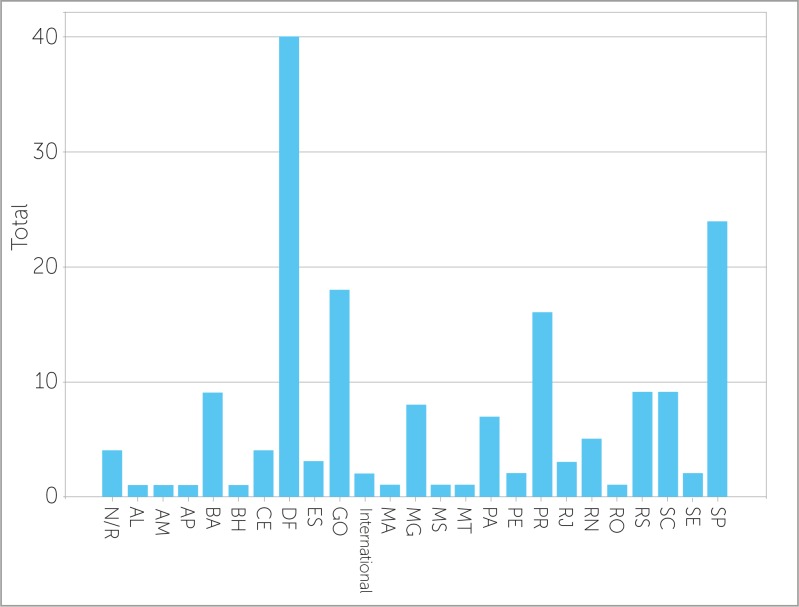
Distribution of answers according to Brazilian states. *N/R - No response.

The answers about where participants acquired TMD knowledge are presented inFigure 3.

**Figure 3 -:**
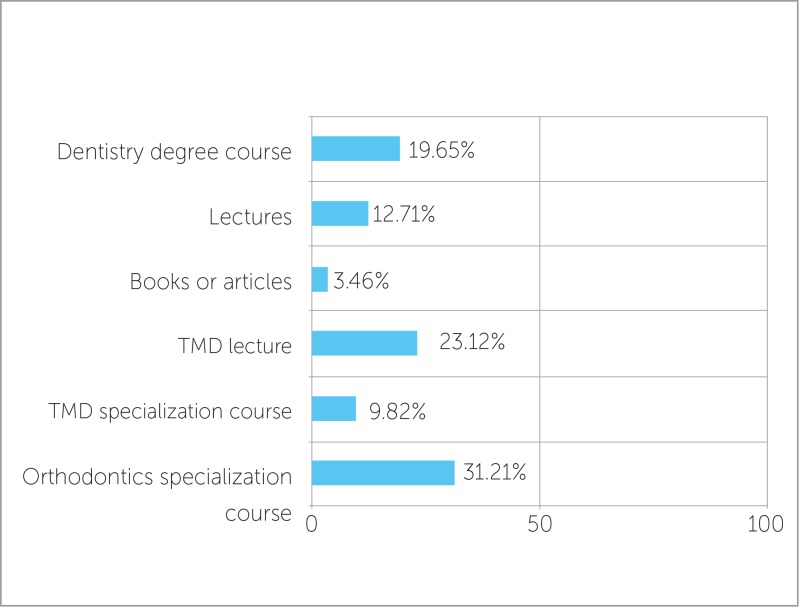
Source of TMD knowledge which served as the basis for other responses.

When analyzing the relationship between TMD treatment and the use of orthodontic appliances, we found that 87.3% of participants had already treated a patient complaining about TMD, whereas 52% had already used orthodontic appliances to treat a TMD patient. Most of those who treated patients with TMD complaint had used orthodontic appliances for this purpose. Thus, a statistically significant association was established (Table 2).

**Table 2 -:** Relationship between treatment of patient complaining of TMD and use of orthodontic appliance for this purpose.

		Have you ever used orthodontic devices to treat TMD symptoms?		
		No	Yes	Total
Have you ever treated a patient with TMD symptoms?	No	21	1	22
	Yes	62	89	151
	Total	83	90	173

P = 0.000.

Answers regarding the statements about whether orthodontic treatment can treat, prevent or cause TMD are presented inTable 3. Most respondents who disagreed with the idea that Orthodontics can treat, cause, or prevent TMD had not used orthodontic appliances to treat TMD symptoms. A statically significant association was found between these statements and the previous use of orthodontic appliances to treat TMD (Table 4).

**Table 3 -:** Answers about whether orthodontic treatment can treat, prevent or cause TMD (Values expressed in %).

	Agree	Disagree	No opinion	Total
Orthodontics is the best treatment option for TMD in patients with skeletal malocclusion	82.1	12.1	5.8	100.0
Orthodontic treatment can prevent the onset of TMD	59.5	37.6	2.9	100.0
Orthodontic treatment can lead to TMD	43.9	52.0	4.0	100.0

**Table 4 -:** Relationship between the answers

	Answer	Have you ever used orthodontic appliances to treat TMD symptoms?		
		No	Yes	Total
Orthodontics is the best treatment option for TMD patients with skeletal malocclusion.*	Disagree	76	66	142
	Agree	3	18	21
	No opinion	4	6	10
	Total	83	90	173
Orthodontic treatment can prevent the onset of TMD.**	Agree	60	43	103
	Disagree	19	46	65
	No opinion	4	1	5
	Total	83	90	173
Orthodontic treatment can lead to TMD.***	Agree	47	29	76
	Disagree	31	59	90
	No opinion	5	2	7
	Total	83	90	173

* P = 0.003;

** P = 0.000;

*** P = 0.001.

## DISCUSSION

The use of questionnaires to collect data presents major limitations, such as poor adhesion of participants, which reduces the number of answers. In a previous study, only 18.30% of participants answered the questionnaire despite control of the number of individuals.^26^ In the present study, we initially sent the questionnaire to Brazilian orthodontists members of the Brazilian Association of Orthodontics (ABOR).Their e-mail addresses are available at ABOR's website, so this was used as source of contact. There was no concern regarding sample selection. Indeed, the authors tried to reach as many Brazilian orthodontists as possible. Thus, the second step was to publish the survey on social networks. Due to lack of criteria on the distribution of the questionnaire, we faced the risk of having non-orthodontists answering it. For this reason, we asked two questions to exclude non-specialists' answers: "How long have you graduated from dental school?" and "How long have you been a specialist in Orthodontics?". Because orthodontic courses last at least 24 months, participants who either answered "less than one year" for the first question or "not orthodontist" for the second, were excluded from the study. Due to lack of exclusivity on sample selection, the result of the present study represents the opinion of specialists that are affiliated to ABOR. This kind of restriction was not an objective of the present study. Our sample probably represents the opinion of those who use the Internet more often, since these people are more likely to answer e-mails and have access to social networks.

The results of this study are in agreement with data obtained by Caldas and Furquim,^27^ particularly with regards to the possibility of orthodontic treatment treating or causing TMD symptoms. On the other hand, when orthodontic treatment was presented as a possible preventive factor for TMD, approximately 59.5% of orthodontists disagreed, differing from the opinion of orthodontists in the Caldas and Furquim's study.^27^ Despite some differences in sampling and methodology , the sentences in both studies were very similar.

Another study^26^ published in 2010 assessed the attitude of a group of Brazilian orthodontists towards diagnosis and management of migraine. The authors concluded that the majority of orthodontists misdiagnosed migraine and mistakenly suggested the use of Orthodontics to treat a concrete clinical case described by the authors. What calls the attention is that most interviewees not only proposed improper procedures, but also tended to indicate some sort of orthodontic treatment.

In our study, the number of orthodontists who stated having treated TMD was high (87.3%), thereby suggesting to be extremely probable that orthodontists are often visited by patients with TMD, which makes TMD knowledge an important matter for the orthodontist. The number of orthodontists who had already used orthodontic appliances to treat TMD is considered high, since it represents 52.02% of the sample in the present study. Even though nearly half of the interviewees answered they have already used some sort of orthodontic appliance to treat TMD, only 12.13% agreed with the statement that "Orthodontics is the best treatment option for TMD in patients with skeletal malocclusion".

We can assume there has been a change in the clinical conduct of these professionals over the years. In other words, former cases treated with orthodontic appliances are no longer seen the same way. Nevertheless, it is possible that these data are indicative that in spite of being aware of current scientific evidence, many orthodontists do not believe in a cause-and-effect relationship between orthodontic treatment and TMD.

A limitation of the present study is the non-assessment of current clinical practice, since questions were only made regarding past activities. This comparison might be interesting in future studies and could indicate whether changes in clinical approaches follow the latest changes in TMD concepts.^25^

The ongoing discussion on scientific evidence has also led to a more careful analysis of the methods used in a few studies. We found out that some studies carried out with appropriate methodology do not show that orthodontic treatment can be effectively used to treat or prevent TMD.^14^^,^ 17 ^,^ 18 ^,^ 19 The studies that show a relationship between malocclusion and signs and symptoms of TMD are among those with the most biases that act as confounding factors, which significantly affect conclusions.^15^^,^ 21 ^,^ 28 ^,^ 29 In addition, an association or a significant correlation does not necessarily imply a cause-and-effect relationship.^15^^,^ 16 ^,^ 30 Furthermore, significant scientific evidence now points to a tendency of non-association among orthodontic treatment, dental occlusion and TMD.^25^^,^ 31

When agreeing with the statement that orthodontic treatment could cause signs and symptoms of TMD, most orthodontists showed they resist to fully accept the non-association of orthodontics and TMD and to include this concept when conducting the clinical diagnosis.

Scientific orthodontic evidence is important for more effective and predictable outcomes. Replacing empirical knowledge with science-based knowledge is part of the scientific maturity process. Nevertheless, this attitude may encounter resistance by some professionals, particularly because the acceptance of a current paradigm implies in abandoning what was once considered true for a significant period of their professional lives. Nonetheless, this resistance may bring harm to patients and to Orthodontics as a whole, particularly when clinicians prescribe less effective or unnecessary treatment, thus not solving patient's issue and affecting Orthodontics credibility as a science. Professionals' opinion was not associated with time since graduation, and goes against the expectation that professionals graduated the longest would be more resistant to breaking a paradigm, thus being more prone to agreeing with an association between TMD and orthodontic treatment.

Answers obtained by means of the questionnaire were more concentrated in the south, southeast and midwest of Brazil, the latter being predominant. Despite this low concentration, it can be considered that the distribution of responses reflects the concentration of professionals in the central-south of the country. The largest concentration in the central region is probably due to the fact that people are more prone to answering questions or to participating in surveys when they know the authors. Furthermore, because the authors are from this area of Brazil, it is highly likely that a higher number of orthodontists from the central region of Brazil were contacted by social networks.

## CONCLUSION

Most orthodontists participating in this study disagree that orthodontic treatment is related with treatment or prevention of TMD, which is in line with the most accepted concept regarding orthodontic treatment and TMD. However, the opinion of most orthodontists differs from current scientific evidence when they say they believe orthodontic treatment could lead to TMD.
